# A Rare Case of Contralateral Ischemic Stroke Recurrence Post-Reperfusion: Successful Management Through Timely Thrombectomy

**DOI:** 10.7759/cureus.54788

**Published:** 2024-02-23

**Authors:** Faisal Althobaiti, Ahmad Alkhatib, Naif Alharbi, Mohammed Alwadai, Ali Alqarni, Khalid Allebdi

**Affiliations:** 1 Neurology, King Fahad General Hospital, Jeddah, SAU; 2 Medicine and Surgery, University of Jeddah, Jeddah, SAU

**Keywords:** nihss (national institutes of health stroke scale), tpa, endovascular thrombectomy, acute stroke, aspect score

## Abstract

Stroke is a predominant contributor to global mortality and disability and represents a complex and heterogeneous disease characterized by diverse risk factors and clinical presentations. The likelihood of stroke patients being at risk of a second stroke within the first five years is higher, especially within the initial two weeks. The distressing prospect of experiencing recurrent stroke shortly after reperfusion therapy adds an additional layer of complexity, potentially reversing prior progress. In the present case, we describe a patient who experienced a recurrent stroke within 24 hours, affecting the contralateral middle cerebral artery (MCA). This recurrence occurred after the individual underwent thrombolysis therapy for the initial stroke, emphasizing the intricate challenges associated with managing such cases and the imperative for targeted interventions to mitigate further risks and enhance patient outcomes.

## Introduction

Ischemic stroke continues to be a substantial contributor to global mortality and persistent disability [1]. Following an initial acute ischemic stroke (AIS), the likelihood of recurrence within 5 years is 24% for females and 42% for males, with the highest risk observed in the initial two weeks following the primary stroke [2]. Recurrent stroke shortly after reperfusion therapy is distressing and has the potential to reverse prior progress. Typically, intravenous thrombolysis is avoided within three months of a stroke due to the increased risk of bleeding. Although evidence from a limited case series suggests its safe and effective use in carefully selected patients, repeated application in early recurrent stroke may pose risks [3].

Timely endovascular thrombectomy (EVT) stands as a potential solution in suitable cases for this challenge. While EVT has a higher recanalization rate for occluded major intracranial arteries [4], instances of recurrent stroke in the same or different cerebral territories, a phenomenon seldom reported during or shortly after thrombolysis, prompts the need for future studies to enhance current guidelines. Here, we present the case of a patient who experienced recurrent stroke within 24 hours in the contralateral middle cerebral artery (MCA) after intravenous (IV) thrombolytic injection.

## Case presentation

**Figure 1:**
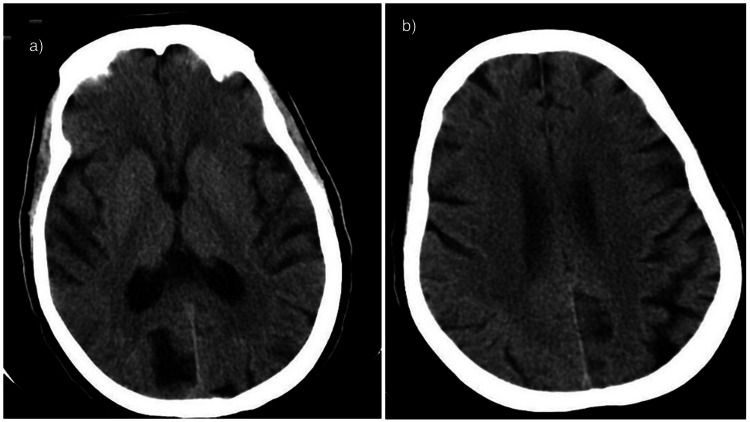
A and B showing the computed tomography (CT) of the brain with a good aspect score

This case involved a 59-year-old woman with a history of diabetes mellitus (DM) who presented within two hours of experiencing sudden right-sided weakness and slurred speech. The patient had no history of headache, nausea, vomiting, trauma, neck manipulation, seizures, loss of consciousness, bleeding from orifices, cardiac disease, fever, or recent infections. On examination, her blood pressure was elevated to 180/90 mmHg, and the random blood glucose levels were 150 mg/dL. The National Institutes of Health Stroke Scale (NIHSS) score was 11, marked by severe aphasia, right facial weakness, and moderate dysarthria (the NIHSS is a reliable, valid, and universal tool for measuring stroke severity) [5]. A computed tomography (CT) scan of the brain showed a good Alberta Stroke Program Early CT (ASPECT) score, which is a scoring system that captures the extent of early infarction based on visually evident hypoattenuation [6], and CT angiography confirmed that there was no evidence of large vessel occlusion (LVO) (Figure 1). The patient met the criteria for tissue plasminogen activator (tPA) treatment and received treatment without immediate complications. This resulted in a decrease in the NIHSS score to 1, with only a mild lower limb drift remaining.

**Figure 2:**
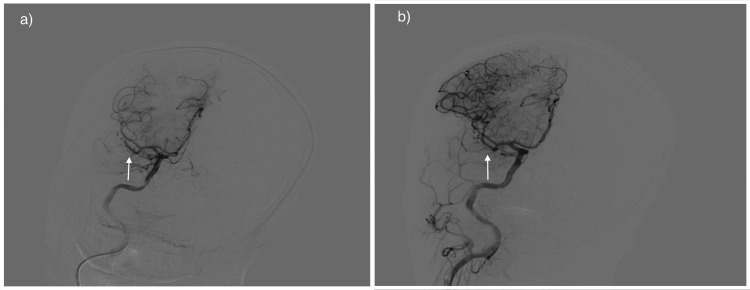
Conventional cerebral angiogram a) Occlusion in the M2 segment of the middle cerebral artery; b) Successful recanalization of the vessel following the thrombectomy procedure

Later that day, the patient’s condition deteriorated, manifesting as a new weakness on the left side of her body, along with aphasia. The NIHSS score increased to 10, marked by the inability to answer questions, severe aphasia, severe dysarthria, left facial partial paralysis, and left upper and lower limb drift, although with limited limb movement confined to hitting the bed. A subsequent CT scan showed no abnormalities, however, the CT angiography revealed an occlusion of the right proximal middle cerebral artery M2 segment. The patient underwent a successful EVT, which resolved the occlusion and improved her clinical status (Figure 2).

**Figure 3:**
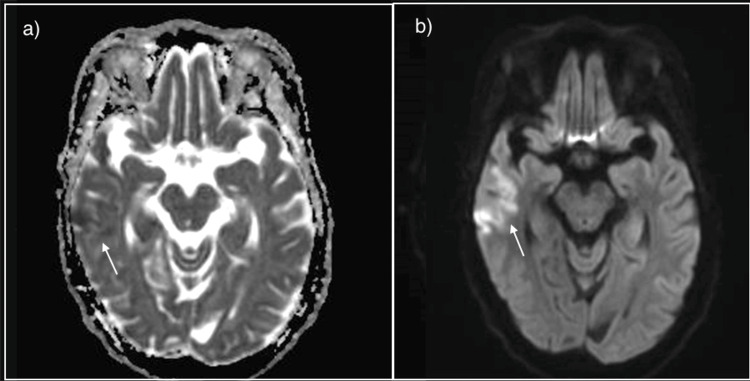
Magnetic resonance imaging of the brain a) low signal on the Apparent Diffusion Coefficient (ADC); b) restricted on the Diffusion-Weighted imaging in the right temporal lobe.

**Figure 4:**
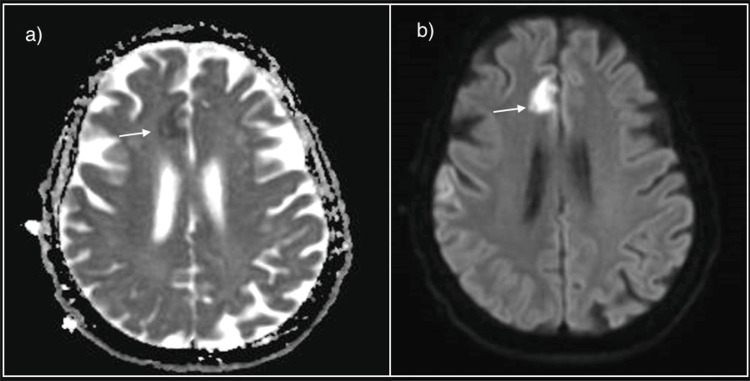
Magnetic resonance imaging of the brain a) low signal on the Apparent Diffusion Coefficient (ADC); b) restricted on the Diffusion-Weighted imaging in the right frontal lobe

Further investigations, including assessments of the HbA1c and lipid profiles, along with autoimmune and thrombophilia workups, revealed no abnormalities. Transthoracic echocardiography revealed diastolic dysfunction, trivial tricuspid regurgitation (TR), and an ejection fraction (EF) of 55%. The transesophageal echocardiography (TEE) did not detect any abnormalities. The patient’s weakness improved within her admission and upon undergoing physiotherapy. Upon discharge, the NIHSS score was 3, indicating mild facial weakness and dysarthria, and magnetic resonance imaging (MRI) revealed acute right temporal and frontal lobe infarcts, along with a left parietal lacunar infarct (Figure 3-4).

## Discussion

In this case, we present the clinical scenario of a patient encountering a recurrent stroke within a remarkably brief timeframe of 24 hours, specifically in the contralateral middle cerebral artery (MCA), subsequent to the administration of an intravenous (IV) thrombolytic injection. This unique and temporally compressed recurrence of stroke events provides a distinctive perspective on the challenges and complexities inherent in the management of AIS, particularly in the context of thrombolytic intervention. Administering timely reperfusion therapy to patients with stroke is critical since the effectiveness of tPA is highly time-dependent. Swift treatment is essential to reduce the mortality risk, prevent symptomatic intracranial hemorrhage, and improve functional outcomes. Our patient demonstrated significant improvement with initial tPA administration, aligned with the current best practice guidelines for acute ischemic stroke management [7]. 

Data from the Oxfordshire Community Stroke Project (OCSP) revealed that the risk of early acute ischemic stroke (ERAIS) recurrence within three months was approximately 15%-20%, with a heightened risk in the initial week. ERAIS is associated with higher mortality and poorer outcomes, influencing the current guidelines for thrombolytic treatment. To adhere to safety protocols, these guidelines exclude a substantial number of ERAIS patients from undergoing repeated intravenous thrombolysis [8,9]. Despite the effectiveness and relative safety of tPA, its use in the treatment of AIS is limited owing to the contraindications associated with intravenous thrombolysis. Present recommendations emphasize that recurrent stroke within three months is a contraindication to thrombolytic treatment, rooted in the presumed increased risk of symptomatic intracerebral hemorrhage (sICH), which has endured without significant alterations over the years. However, some recently published case series showed the marginal effects of using tPA in early ischemic strokes without significant complications or worse outcomes [10-13]. Svjetlana et al. reported a patient with ERAIS within 54 hours of repeated tPA in combination with repeated mechanical thrombectomy with satisfactory functional recovery [14]. 

In recent clinical trials and treatment recommendations for ERAIS, mechanical thrombectomy has been established to be superior to systemic thrombolysis alone, particularly in patients with large-vessel occlusion (LVO). In this case, occlusion of the right MCA was identified. Consequently, we opted not to administer tPA, proceeding promptly with mechanical thrombectomy. This decision aligns with the current understanding that mechanical thrombectomy is particularly effective in patients with an LVO. Our hospital, a comprehensive stroke center with readily available neurointerventionalists, facilitated this swift and targeted intervention [15,16].

There is a limited body of literature discussing the repeated use of mechanical thrombectomy in patients with acute stroke, with most cases focusing on recurrent strokes in the same hemisphere occurring more than 24 hours apart [17]. This differs from our patient’s case, wherein she experienced a very early recurrent ischemic stroke, notably in a different hemisphere. The rate of early recurrence in cerebral infarction is contingent on the etiology of stroke, particularly in cardioembolic strokes, which exhibit an approximately 7% recurrence risk within the first two weeks, with a mean time to recurrence of 12 days [18]. A report by Quintas et al. highlighted that reocclusion occurred just 28 hours after the first recanalization procedure in a patient with atrial fibrillation [19]. Early recurrence of thromboembolic events has been observed in patients with prothrombotic states related to malignant processes. Despite a thorough investigation, the mechanism underlying the early stroke recurrence in our patient remains unclear. Presently, it is presumed to be a cryptogenic stroke, more specifically an embolic stroke of undetermined etiology (ESUS), owing to its distinct stroke pattern and the absence of identified alternative causes, a categorization consistent with the prevailing stroke literature [20].

## Conclusions

This case highlights the intricate nature of managing strokes and underscores the significance of ongoing evaluation. It emphasizes the crucial role that comprehensive diagnostic imaging and intervention play in effectively addressing occlusions and improving the clinical status of stroke patients. Additionally, regular monitoring and assessment are crucial for the early detection of stroke recurrence and for fostering patient outcomes.
